# The molecular chaperon AKR2A increases the mulberry chilling-tolerant capacity by maintaining SOD activity and unsaturated fatty acids composition

**DOI:** 10.1038/s41598-018-30379-9

**Published:** 2018-08-14

**Authors:** Lin Chen, Yuqi Hou, Wenjun Hu, Xiaoyun Qiu, Hongling Lu, Jia Wei, Shaofang Yu, NingJia He, Hong Zhang, Guoxin Shen

**Affiliations:** 1 Sericultural Research Institute, Zhejiang Academy of Agricultural Sciences, Hangzhou, 310021 China; 2 Department of Biological Sciences, Texas Tech University, Lubbock, Texas 79409 USA; 3 State Key Laboratory of Silkworm Genome Biology, Southwest University, Chongqing, 400715 China

## Abstract

Chilling is common in nature and can damage most plant species, particularly young leaves and buds. Mulberry (*Morus* spp.) is an economically important food source for the domesticated silkworm (*Bombyx mori*). However, weather and climatic extremes, such as “late spring coldness”, seriously damage mulberry buds and young leaves. The molecular mechanism involved in the differing mulberry chilling tolerance is unclear. In the present study, we found that mSOD1, mFADII, and mKCS1 interacted with mAKR2A and that the expression of mAKR2A, mSOD, mFAD, and mKCS1 in the chilling-tolerant mulberry variety was higher than that in the chilling-sensitive variety. Unsaturated fatty acids content and superoxide dismutase (SOD) activity in the chilling-tolerant variety was higher than that in the chilling-sensitive variety. After chilling treatment, mSOD1, mKCS1 and mAKR2A expression in the chilling-tolerant variety was reduced to lower than that in the chilling-sensitive variety, whereas mFADII expression increased in the chilling-tolerant variety compared with that in the chilling-sensitive variety, suggesting that the increased expression of the molecular chaperon mAKR2A helped to maintain or prompted the chilling-related proteins in the chilling-tolerant variety.

## Introduction

Temperature is one of the primary environmental factors affecting plant geographic distribution. Chilling conditions defined as temperatures ranging from 20 °C–0 °C, are common in nature and impose a major environmental restriction on plant performance, especially in cold climates at high latitudes or altitudes^1^. As an abiotic stress, chilling can damage most plant species. Plants have evolved several physiological and molecular adaptations to increase their chilling-tolerant ability. Studying the molecular mechanisms regulating the cold responses of plants will help understand plant adaptation to local environments at the molecular level. Many plants show increased freezing tolerance on exposure to low nonfreezing temperatures, known as cold acclimation.

Mulberry (*Morus spp*.) is a deciduous perennial tree and an economically important food source for the domesticated silkworm (*Bombyx mori*)^2^. Mulberry is widely planted in the Eurasian continent, Africa and America. The family Moraceae comprises 37 genera and approximately 1,100 species, including well-known plants such as mulberry, bread-fruit, fig, banyan and upas^3^. The Moraceae resources are rich in China, including 11 species and 12 cultivars throughout all over the country^4^. Some mulberry plants have important economic and medicinal value. Utilization of the mulberry-silkworm interaction began at least 5,000 years ago. As the only food material available for the silkworm, mulberry has a long cultivation history in China, Japan, India and several other Asian countries^5^. Genome sequencing of mulberry was completed in 2010 and will facilitate the improvement of mulberry, because little information was available regarding the molecular biology. In recent decades, weather and climatic extremes have had seriously damaging effects on sericulture in China. “Late spring coldness”, which is a sudden low temperature during the spring warming, results in mulberry buds and young leaves freezing to death. The chilling-tolerant of mulberry plants varies greatly, but the molecular mechanism to explain the difference in cold tolerance remains unclear. Clarification of the molecular basis of chilling-tolerant in mulberry may allow selection and breeding of chilling-tolerant mulberry cultivars, and expansion of the cultivated mulberry planting area.

**Figure 1:**
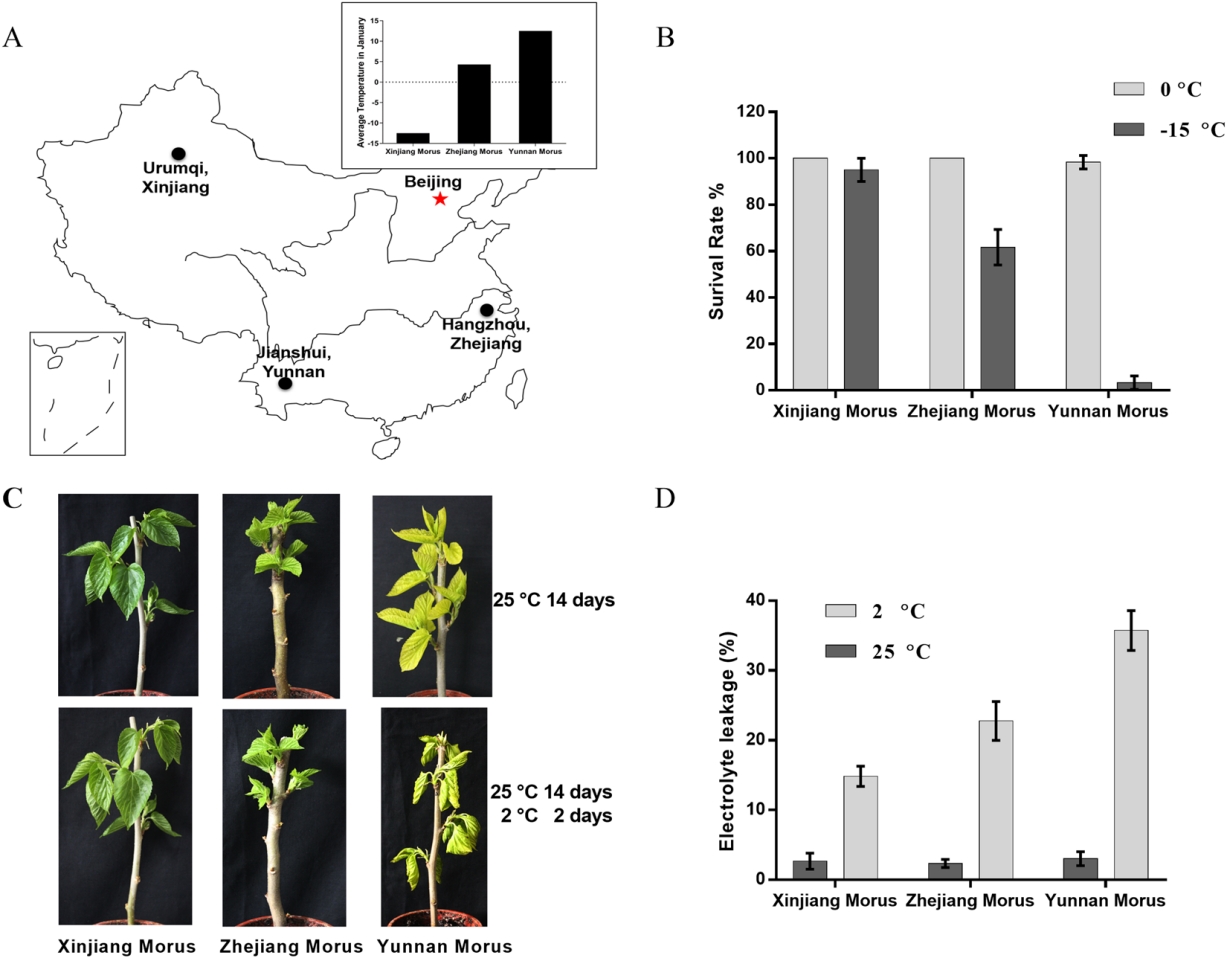
Cold tolerance assays. (**A**) The location of the three mulberry varieties in China (This map was generated by Microsoft Office, https://products.office.com/zh-cn/compare-all-microsoft-office-products?tab=1). The average January temperature is shown in the inner figure. (**B**) Low temperature assays. The mulberry shoots in the dormancy stage were cut and stored at 0 °C and −15 °C for 30 days. The shoots were then transferred to 25 °C to monitor the mulberry burgeon ratio. (**C**) Chilling stress assays. Shoots with 14 days old leaves, grown at 25 °C, were transferred to 2 °C for 2 days. (**D**) Electrolyte leakage assay.

Ankyrin-repeat containing protein 2 A (AKR2A) is an essential molecular chaperone that binds to hydrophobic amino acid residues, which prevents membrane proteins from aggregating after translation in the cytoplasm^6–8^. *akr2a* mutants display a chilling-sensitive phenotype, suggesting that *AKR2A* plays important roles in the plant network responding to cold stress. Molecular analyses of *akr2a* mutants and AKR2A knockout lines indicate that AKR2A is essential for plant growth and development^8,9^. The AKR2A of mulberry (mAKR2A) shows 65% similarity with the AKR2A of Arabidopsis (atAKR2A), with a highly conserved Ankyrin domain (supplemented data Fig. 1), suggesting that mAKR2A may play an important role in the mulberry cold tolerance network. In a previous study, AKR2A recognized a single transmembrane domain followed by one or a few positively charged amino acid residues. However, in the present study, AKR2A also interacted with proteins without the ABS sequence, suggesting that AKR2A has more functions during plant growth and development.

Using yeast-two-hybrid (Y2H) library screening, mSOD1, mFADII, and mKCS1 were found to interact with mAKR2A. In the present study, the expression level of *mAKR2A*, *mSOD*, *mFADII*, and *mKCS1* in the chilling-tolerant mulberry variety (Xinjiang Morus) was higher than that in the chilling-sensitive variety (Yunnan Morus). The SOD activity and unsaturated fatty acid content in the chilling-tolerant variety, corresponding with the gene expression results, were higher than those in the chilling-sensitive variety. After chilling treatment, mSOD1 and mKCS1 mAKR2A expression in the chilling-tolerant variety was reduced to approximately 30%, whereas in the chilling-sensitive variety, the reduction was 70%. After chilling treatment, mFADII expression increased approximately 1-fold in the chilling-tolerant variety, whereas the chilling-sensitive variety only showed an increase of 0.4-fold. These results suggested that the highly expressed molecular chaperon mAKR2A helped to maintain or prompted cold-related gene expression in the chilling-tolerant mulberry variety.

## Results

### Chilling-tolerant differences in three-natural mulberry varieties

The three mulberry varieties used in this study were collected in China and selected such that their habitats covered areas with the coldest average temperature in January at Urumqi, Xinjiang Uygur Autonomous Region (Xinjiang Morus), the warmest temperature at Jianshui-Xian, Yunnan Province (Yunnan Morus), and intermediate temperatures at Hangzhou, Zhejiang Province (Zhejiang Morus). Xinjiang Morus was located northernmost among the populations with the lowest monthly average temperature in January, whereas Yunnan Morus experienced the highest monthly average temperature in January. The distribution map and the correlated average temperature for January information are shown in Fig. 1A.

To investigate the cold tolerance differences among the three populations, the survival capacity of the mulberry plants was determined after treatment under low temperature. The survival rate of all three mulberry plants was high under at 4 °C. At temperatures below −15 °C, the Yunnan Morus showed a significant reduction in survival rate (more than 90% died), the Zhejiang Morus showed a 45% reduction in survival rate, whereas the survival rates of Xinjiang Morus were not affected. These results suggested that the cold tolerance of Xinjiang Morus was the highest among the three varieties, and that Yunnan Morus was the most sensitive to low temperature (Fig. 1B).

To evaluate the chilling tolerance of these three varieties under “late cold spring” conditions, the new buddings (14 days old) grown under a normal condition (25 °C) were transferred to cold conditions. After 2 days of treatment, the leaves of Yunnan Morus withered, whereas the leaves of Xinjiang Morus and Zhejiang Morus were not seriously affected (Fig. 1C).

To determine the chilling damage of the three mulberry varieties, the electrolyte leakage assay was performed. Under normal growth conditions, the EL values of Xinjiang Morus, Zhejiang Morus and Yunnan Morus were approximately 3% (Fig. 1D), indicating no difference among the three mulberries under the normal condition. After chilling stress treatment, the EL values increased in all three varieties, however, the EL of Yunnan Morus was obviously higher than that of Zhejiang Morus, and the lowest EL was observed in Xinjiang Morus (Fig. 1D). Xinjiang Morus most likely had greater ability to maintain membrane integrity under chilling stress than that of the other varieties.

### ROS production and SOD detection in whole leaves

**Figure 2:**
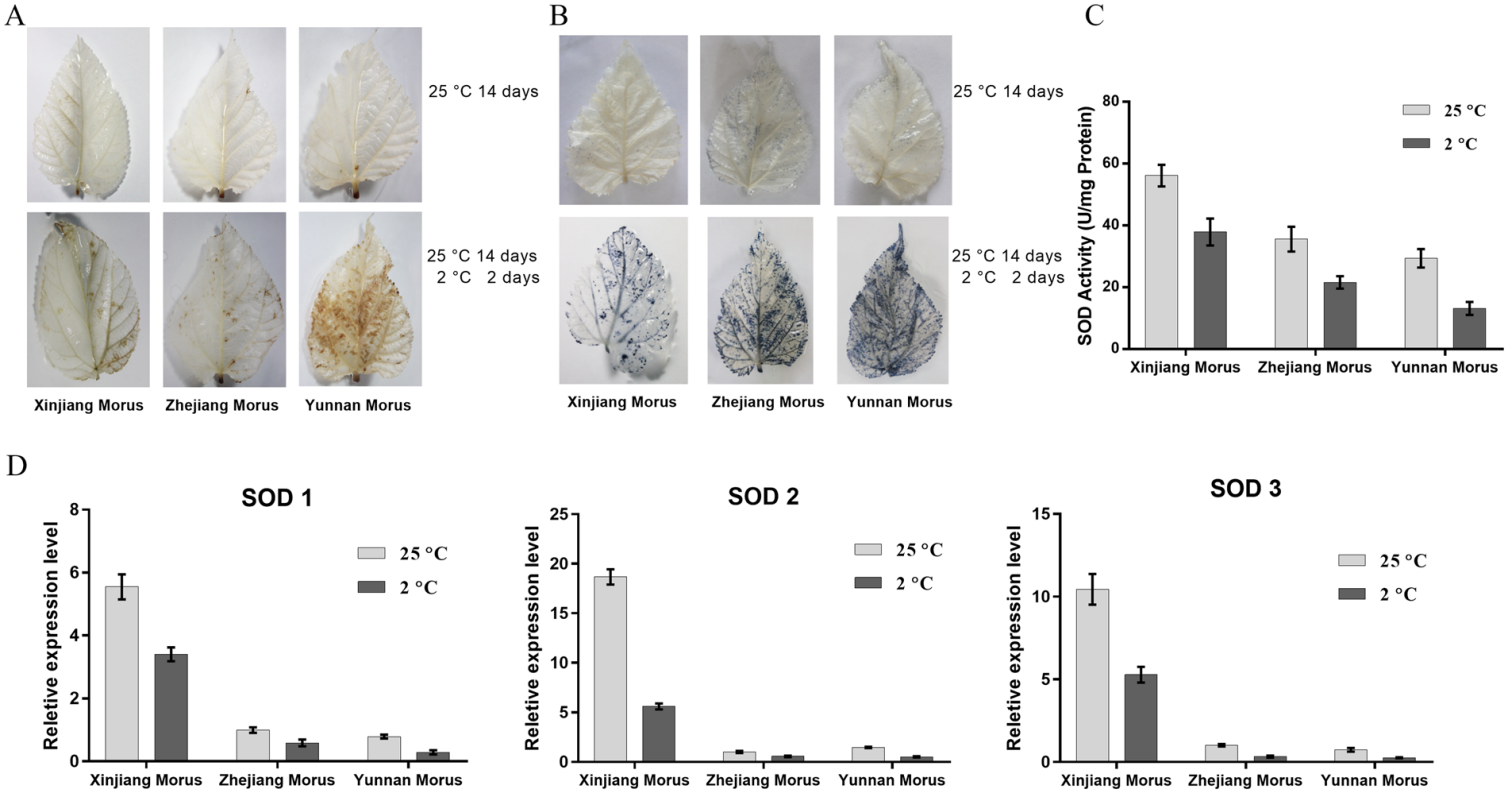
Reactive oxygen species (ROS) detection. (**A**) H_2_O_2_ accumulation under chilling conditions was visualized by DAB staining in mulberry leaves. (**B**) ${{\rm{O}}}_{2}^{\bullet -}$ generation was visualized by NBT staining as blue coloration in mulberry leaves. (**C**) Detection of SOD activity in the mulberry leaves. (**D**) Analysis of SOD-related gene expression in the three mulberry varieties.

During chilling stress, reactive oxygen species (ROS) increase suddenly in plants. ROS are toxic to proteins, DNA and lipids, and scavenging ROS at the proper site and time is important. In the present study, we used two histochemical staining approaches, DAB and NBT, to detect ROS production in whole mulberry leaves. The DAB staining results demonstrated that more H_2_O_2_ accumulated in Yunnan Morus leaves in cold conditions than that in Zhejiang Morus and Xinjiang Morus, which had less H_2_O_2_ detected in leaves. NBT staining illustrated a trend similar to that of the DAB results. Cold conditions induced less ${{\rm{O}}}_{2}^{\bullet -}$ accumulation in Xinjiang Morus, whereas increased ${{\rm{O}}}_{2}^{\bullet -}$ accumulation was detected in Zhejiang Morus leaves, and the ${{\rm{O}}}_{2}^{\bullet -}$ content in Yunnan Morus leaves was the highest. These results indicated that Yunnan Morus was the most sensitive to chilling among the three-tested mulberry varieties (Fig. 2A,B).

A balance occurs between ROS production and scavenging. Chilling-induced ROS is likely cleared in chilling-tolerant plants by high antioxidant enzyme activity. The DAB and NBT staining results suggested that SOD activity was higher in Xinjiang Morus than that in Zhejiang Morus or Yunnan Morus. The increase in SOD activity was 2-fold higher in Xinjiang Morus than that of the Yunnan Morus. The SOD activity of Zhejiang Morus was between that of Xinjiang Morus and Yunnan Morus before cold treatment. After cold treatment, the SOD activity of Xinjiang Morus reduced 20%, whereas the SOD activity of Zhejiang Morus or Yunnan Morus was only approximately half of that before the cold treatment (Fig. 2C).

The expression levels of three SOD family genes in mulberry *mSOD1*, *mSOD2*, and *mSOD3* were analyzed. The same trend was observed in the expression of all three *SOD* genes under cold assays. The expression of the three genes was reduced under chilling conditions in the three mulberry varieties. The *SOD* expression level in Xinjiang Morus was the highest among the three varieties before the chilling treatment. After the chilling assays, the *SOD* expression level in Xinjiang Morus was much higher than that of Zhejiang Morus or Yunnan Morus (Fig. 2D). This result was consistent with those of the SOD activity assays and DAB staining results.

### Fatty acids composition analysis

**Figure 3:**
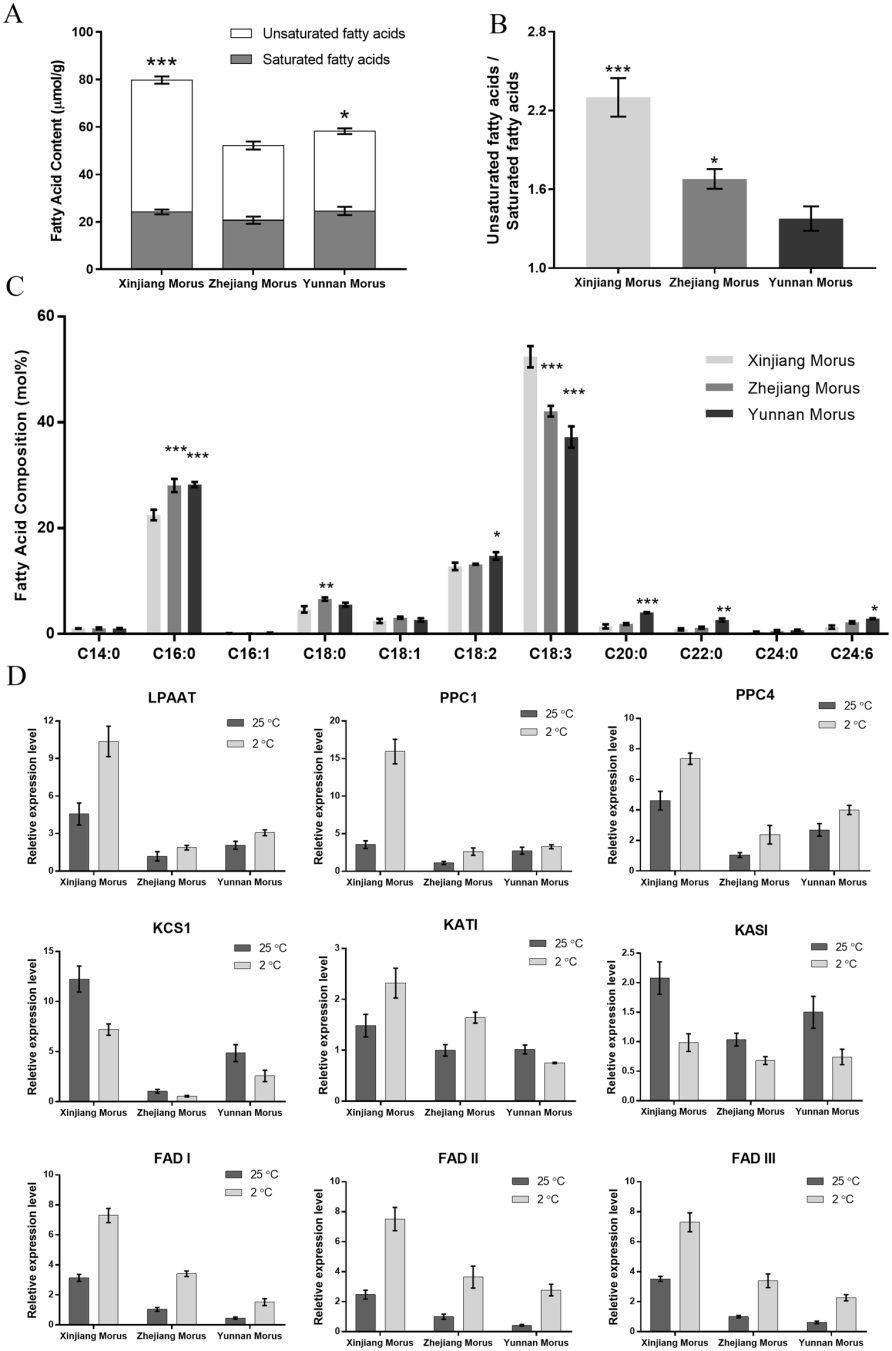
Fatty acid content and fatty acid related gene expression. (**A**) Fatty acid content analyses of the three mulberry varieties. (**B**) The ratio of unsaturated fatty acids to saturated fatty acids. (**C**) Fatty acid composition analyses of the three mulberry varieties. Statistical significance was determined using one-way analysis. ^*^< 0.05, ^**^P < 0.01, ^***^P < 0.001. (**D**) Analysis of the expression level of fatty acid related genes.

Cell membrane lipids transform from a liquid- crystalline phase to a gel phase under chilling stress, and this transition impairs membrane permeability, resulting in plant growth disorders and even plant death. Many studies demonstrate that cold tolerance is related to the composition of plant membrane lipids. The increase in unsaturated fatty acids content is likely an important factor in plant adaptation to cold stress^10–13^. To investigate the correlation between chilling tolerance and the fatty acids composition of mulberry leaves, leaf fatty acids composition of the three mulberry varieties was analyzed. The results showed that Xinjiang Morus had the highest fatty acids content among the three mulberry varieties. The fatty acids content of Yunnan Morus was lower than that of Xinjiang Morus but greater than that of Zhejiang Morus (Fig. 3A). Very high levels of fatty acids unsaturation are commonly observed in chilling-tolerant plants. The results of the fatty acids composition analysis showed that Xinjiang Morus had the highest unsaturated fatty acids ratio (Fig. 3B), whereas the unsaturated fatty acids ratio of Yunnan Morus was the lowest among the three varieties. Further analyses showed that the levels of saturated fatty acids (C16:0, C18:0, C20:0, C22:0, and C24:0) in Yunnan Morus were high, whereas the levels of unsaturated fatty acids, paricularly C18:3, were significantly lower than those in Xinjiang Morus or Zhejiang Morus (Fig. 3C).

To examine the response of mulberry fatty acids genes to chilling conditions, the expression levels of genes involved in fatty acids biosynthesis were analyzed (Fig. 3D). The results showed that *LPAAT*, *PPC1*, and *PPC4*, which are important for the fatty acid biosynthesis, were highly expressed in Xinjiang Morus, and the lowest levels were detected in Zhejiang Morus. The results were consistent with the results of fatty acids content. The expression level of *FADI*, *FADII*, and *FADIII*, which are essential for fatty acids desaturation, was higher in Xinjiang Morus than that in Zhejiang Morus or Yunnan Morus. *FAD* expression levels in Zhejiang Morus were higher than those in Yunnan Morus. These results were consistent with the unsaturated fatty acids ratios of the three mulberry varieties (Fig. 3B). After the chilling assays, the expression of *LPAAT*, *PPC1*, *PPC4*, *FADI*, *FADII*, and *FADIII* was up regulated, indicating improvement of fatty acids content, particularly the unsaturation levels of fatty acids, was an important cellular response for mulberry. The very-long chain fatty acids biosynthesis enzyme *KCS1* was down regulated under chilling conditions. The highest expression level of *KCS1* was detected in the Xinjiang Morus, while the intermediate and lowest expression levels of KCS1 were in Yunnan and Zhejiang Morus, respectively. Although the fatty acids component, viz., C20:0, C22:1, C20:0 and C24:6, had very low expression (<5 mol%) among the three different varieties, the content expressed was higher in the Yunnan variety than that in the other two varieties studied. The high expression level of *KCS1* in Yunnan Morus was consistent with the fatty acids composition results showing high content of C20:0, C22:0, and C24:0 compared with that in Xinjiang Morus and Zhejiang Morus.

### Molecular chaperon mAKR2A interacts with mSOD1, mKCS1 and mFADII

**Figure 4:**
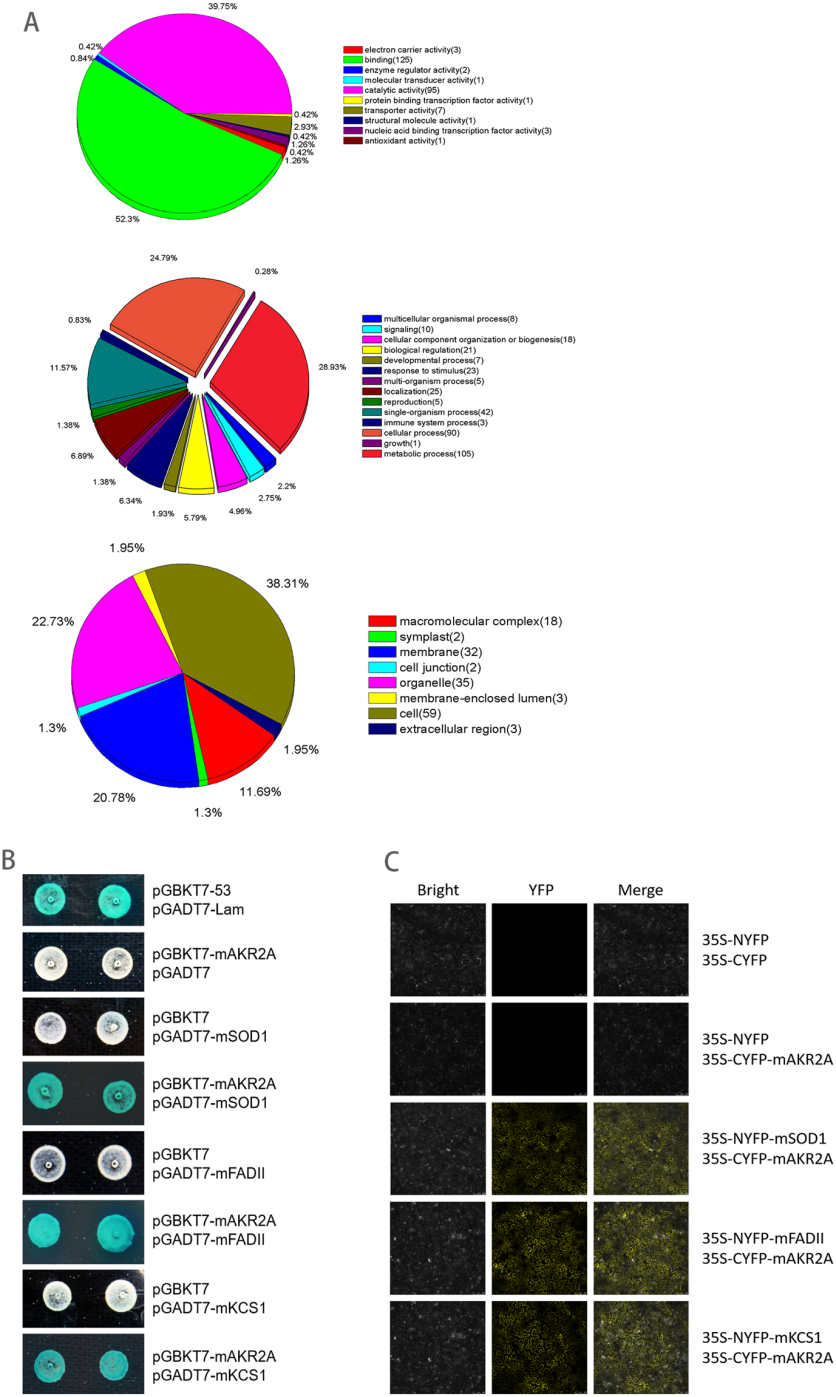
Protein interactions of mAKR2A with mSOD1, mKCS1 or mFADII. The interactions of mAKR2A with mSOD1, mKCS1 or mFADII were analyzed by the yeast two-hybrid assay (**A**) and BiFC analysis (**B**). Empty vectors were used as negative control.

The mulberry yeast library construction and mating assays weres performed according to the Yeastmaker Yeast Transformation protocol (Clontech). In total, 934 blue colonies were identified and sequenced, and 296 mulberry genes were characterized after removing the duplicated data, and 291 homologues of these genes were characterized in Arabidopsis. Functional classification of the identified proteins was performed by Blast2go analysis (Fig. 4A). Their cellular component association, molecular function, and biological processes were analyzed, and 210 annotated peptides were obtained. However, our recent Yeast-2-Hybrid experiment demonstrated that aAKR2A interacted with both membrane proteins (such as FADII and KCS1) and cytoplasmic proteins (such as SOD1).

To determine the interaction between mAKR2A and mSOD1, mKCS1, and mFADII, Y2H and BIFC assays were performed. Previous studies demonstrated that AKR2A binds specifically to a transmembrane domain followed by a few positively charged amino acid residues, and this sequence is named the AKR2A-binding sequence (ABS). mKCS1 and mFAD2 contain the ABS motif. However, the transmembrane domain was not detected in mSOD1. Residues 1 to 200 of mAKR2A were used as prey with the full-length proteins of mSOD1, mKCS1, and mFAD2 used as baits. The results showed that mAKR2A interacted with mSOD1, mKCS1, and mFADII *in vitro* (Fig. 4B). BIFC assays were then performed for further visualization of protein-protein interaction *in vivo*. The BIFC results demonstrated that mAKR2A interacted with mSOD1, mKCS1, and mFADII (Fig. 4C). These results indicated that AKR2A could interact with a protein without the ABS.

### mAKR2A regulates the expression level of the interacting proteins

**Figure 5:**
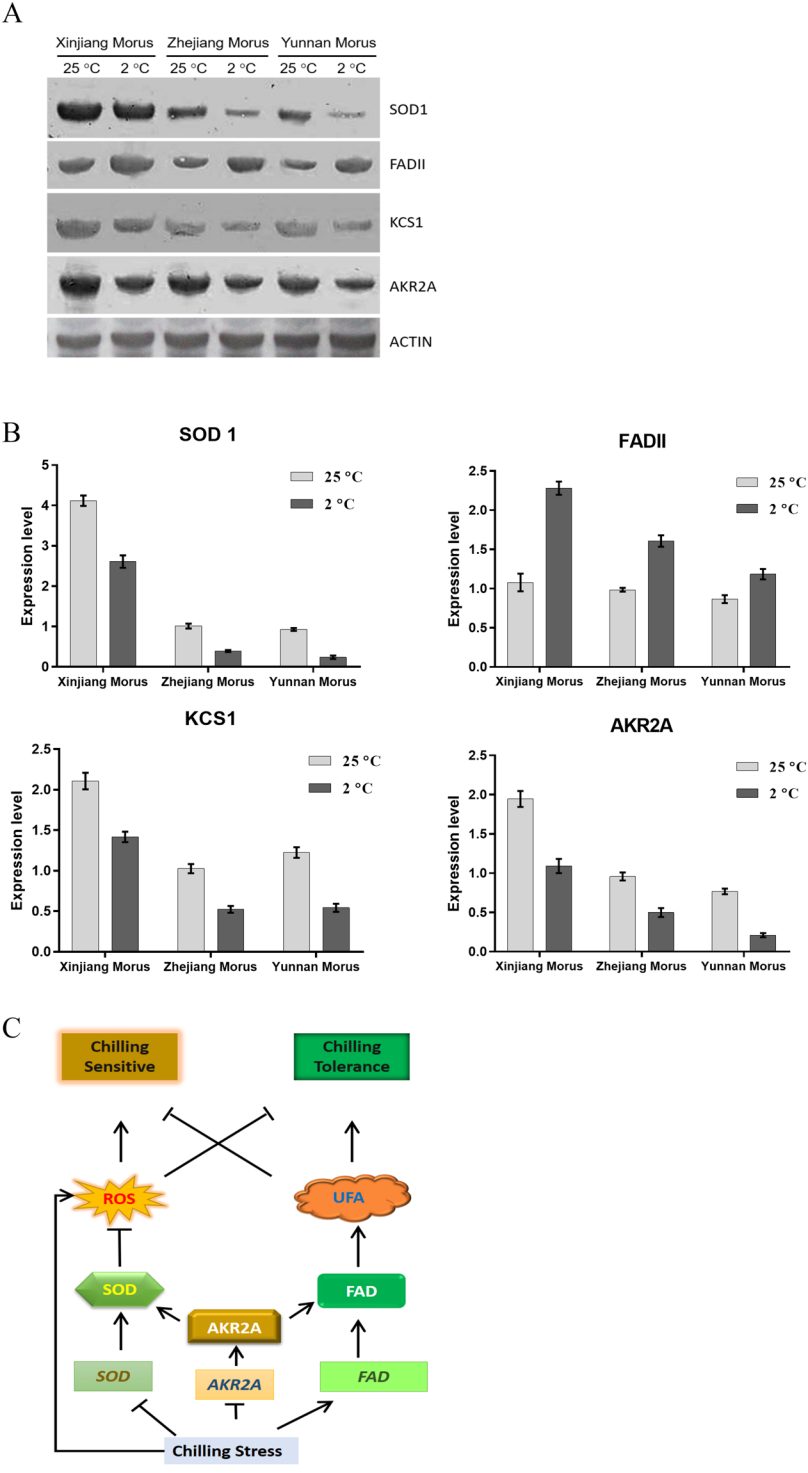
Expression analysis of mAKR2A, mSOD1, mKCS1 and mFADII proteins in mulberry leaves. (**A**) Total leaf protein extracts were prepared from 20-day-old plants under normal or chilling conditions and analyzed by SDS-PAGE and immunoblotting using anti-AKR2A, anti-SOD1, anti-FADII, anti-KCS1 or anti-Actin antibodies, as indicated. N, normal condition, 25 °C for 14 days; C, chilling condition, 25 °C for 14 days and 2 °C for 2 days. (**B**) Analysis of SOD1, FADII, KCS1 and AKR2A expression levels. The expression level data from A were analyzed using ImageJ (http://rsb. info. nih. gov/ij/). (**C**) The proposed mulberry chilling-resistance model. Cold stress induces *FADII* expression and reduces *AKR2A* and *SOD1* expression. AKR2A positively regulates the expression of SOD1 and FADII. SOD1 can increase the plant chilling resistance by clearing the ROS induced by the chilling stress. FADII improves the plant chilling resistance by increasing the unsaturated fatty acid composition. Arrows denote positive effects, and bars indicate negative roles.

The highest expression level of mAKR2A among the three varieties was detected in Xinjiang Morus, and lower levels were observed with Zhejiang and Yunnan varieties, in non-stressed control plants. This result consistent with the results of the chilling tolerance assays. However, the reduction in the expression level of mAKR2A in the chilling stressed plants was greater in the chilling sensitive varieties than the chilling tolerant one (Fig. 5A).

The Western blot results showed that the expression of mFADII increased after chilling treatment. The expression of mFADII of Xinjiang Morus increased by approximately 1.2-fold and that in Zhejiang Morus and Yunnan Morus increased by 0.6-fold and 0.4-fold, respectively. The expression of mSOD1 and mKCS1 was reduced under chilling conditions. The expression level of mSOD1 decreased by 40%, 60%, and 70% for Xinjiang Morus, Zhejiang Morus, and Yunnan Morus, respectively, whereas the expression of mKCS1 was reduced 70%, 50%, and 40% in Xinjiang Morus, Zhejiang Morus, and Yunnan Morus, respectively, compared with that before the chilling assays. The results showed that the expression levels of mFADII, mSOD1, and mKCS1 in Xinjiang Morus were the highest among the three mulberry varieties. The Xinjiang Morus was the chilling-tolerant mulberry with the highest mAKR2A expression level, whereas the lowest mAKR2A were detected in the chilling-sensitive mulberry, Yunnan Morus. The expression of mFADII, mSOD1, and mKCS1 in Yunnan Morus was also the lowest under chilling stress (Fig. 5A,B).

Overall, the results suggested that the transcription of SOD1 was reduced under chilling stress resulting in decreased SOD1 translation. Under chilling stress, when the ROS in cells not scavenged in time, plant injury occurs due to accumulation of high ROS in plant cells. However, fatty acid biosynthesis (*LPAAT*, *PPC1*, and *PPC4*) and desaturation genes (*FADII*) were up-regulated under chilling stress, indicating that increased fatty acids content, particularly the desaturated fatty acids ratios was important for plants to fight chilling stress. The molecular chaperon AKR2A helped the expression of chilling-response genes. High expression of AKR2A implied high expression of SOD1, FADII, and LPAAT, resulting in low ROS and high content of unsaturated fatty acids, further resulting in high chilling-tolerance capacity (Fig. 5C).

## Discussion

Temperature is one of the major abiotic factors that affect the geographical distribution of wild and cultivated plants. Many tropical and subtropical plants, such as citrus and tea bushes, are limited for growth in some parts of the world according to their cold tolerance. Chilling conditions, defined as 0 −15 °C, are a major environmental restriction on plant performance in alternating warm and cold seasons, particularly in the late spring. Elucidation of the mechanism of chilling tolerance is the prerequisite to enhance the selection procedure of breeding targeted at developing chilling-tolerant high yielding cultivars.

Comparing the fatty acids of cellular membranes, more unsaturated fatty acids are detected in chilling-resistant plants than in those that are the chilling sensitive^11,14,15^. Cellular membranes are disrupted under chilling injury, and the degree of unsaturated fatty acids (UFAs) is positively correlated with chilling injury. Therefore, the proportion of unsaturated fatty acids may allow membranes to remain fluidic in nature instead of crystallizing and provides protection against damage from chilling temperatures. When exposed to cold temperatures, a great number of genes are involved in the different steps of chilling stress responses^16–18^. In the present study, the genes of fatty acids bio-synthesis and fatty acids desaturation were up regulated under chilling conditions. LPAAT, an essential acyltransferase involved in membrane-lipid biosynthesis, controls the conversion of 1-acyl-sn-glycerol-3-phosphate to phosphatidic acid (PA) and mediates membrane synthesis^19–21^. PPC1 and PPC4 are ubiquitous plant proteins involved in the tricarboxylic acid cycle, which plays a central role in generating carbon skeletons for biosynthetic processes such as membrane biosynthetic metabolism^22–24^. The expression levels of LPAAT, PPC1, and PPC4 increased under chilling conditions in the three mulberry varieties, indicating that improving the fatty acids content is a conserved strategy in mulberry. PPC1 and PPC4 are also involved in the adaption of plants to abiotic stress^22^, suggesting that increased expression of PPC1 and PPC4 could help the mulberry plant to resist chilling stress.

Fatty acids with 20 or more chain carbons are called very long chain fatty acids (VLCFAs) that are synthesized through the elongation of C16 or C18 fatty acids by the 3-Ketoacyl-CoA Synthase (KCS) system^25,26^. In plants, VLCFAs are implicated in a several cellular processes, including the formation of cuticular waxes and cellular membranes, and trafficking of lipids and proteins^27^. The expression level of mulberry KCS1 decreased under chilling conditions, indicating that the production of VLCFAs was not a quick-response method in mulberry chilling resistance. However, the expression of FADs was up regulated suggesting that increased the expression of fatty acids desaturation genes was induced by low temperature, and that the unsaturated fatty acids content was important for mulberry chilling resistance. FADs play important roles in plant responses to abiotic stresses, and unsaturated fatty acids are an important factor contributing to cold tolerance in plants^28,29^. The results showed that the chilling resistant mulberry, Xinjiang Morus, had a higher level of mFADII expression, whereas the expression level of FADs in Yunnan Morus (chilling-sensitive) was low regardless of the chilling treatment. Environmental diversity likely led to the different responses of these mulberry plants to chilling. The Xinjiang Morus grows at high latitude areas and experiences the lowest environmental temperatures among the three varieties. The unsaturated fatty acids ratio is well known to increase by the regulated activity of fatty acids desaturases in cold regions^12,13^. Consistent with previous studies, the primary difference observed in fatty acid composition was the accumulation of the trienoic unsaturated fatty acid, C18:3. The ratio of C18:3 was associated with chilling resistance in all three varieties. The Xinjiang Morus had the highest content of C18:3 and approximately 55% of the total cellular fatty acids was C18:3. The C18:3 content in Zhejiang Morus was 42%, wheras that in Yunnan Morus was 37%. Very long-chain fatty acids (VLCFAs) and their derivatives are the main constituents of cuticular waxes that prevent disease at the leaf surface. Considering that Yunnan Morus grows in subtropical areas, the highest VLCFAs ratio might help to protect the Yunnan Morus from the diverse pathogens in its environment^30,31^.

Another effect of chilling stress is the increase in ROS production. The expression level of ROS-scavenging enzymes is very important for plant chilling resistance. Our results showed that the expression levels of *SOD* genes were in consistent with the chilling tolerance of the three mulberry varieties and decreased under chilling conditions. This gene expression pattern matched the SOD enzymatic activity. The ROS scavenging rate during the chilling stresses was related to the expression level of the SOD gene. The high expression level of SOD could easily scavenge the overproduction of ROS. The SOD expression and activity results were in consistent with the mulberry chilling resistance.

Most newly synthesized proteins must fold to unique three-dimensional structures to become functionally active. Molecular chaperones protect the newly synthesized protein chains from misfolding and aggregation, and help them to efficiently reach their native state or location^32,33^. AKR2A is a newly identified molecular chaperon that prevents the newly synthesized proteins from forming aggregates after translation^8,9,34^. The expression of AKR2A was reduced under chilling conditions, suggesting that AKR2A is not a chilling-responsive protein. With the highest expression of mAKR2A in Xinjiang Morus, the degree of reduction of mSOD1 (26.5%) and mKCS1 (30%) expression was the lowest among the three varieties under chilling conditions, compared with 29.7% and 38.6% in Zhejiang Morus and 37.7% and 46.3% in Yunnan Morus were, respectively. mFADII expression increased by 1.15-fold in Xinjiang Morus, wheras the Zhejiang Morus and Yunnan Morus showed an increase of 0.6-fold and 0.45-fold. The results indicated that the expression level of mAKR2A was related to that of mFADII, mSOD1, and mKCS1. High levels of mAKR2A expression could maintain the expression of the proteins at high levels to improve mulberry chilling resistance. mAKR2A binding sequences were detected in mFADII and mKCS1, but the ABS was not found in mSOD1. The interaction between mSOD1 and mAKR2A suggested that mAKR2A interacted with transmembrane proteins as well as cytoplasmic proteins, and that mAKR2A could play different roles that are yet to be discovered.

## Materials and Methods

### Plant material

Three mulberry varieties were used in the study. Xinjiang Morus plants were collected from Urumqi, Xinjiang Uygur Autonomous Region, China. Zhejiang Morus plants were collected from Hanghzou, Zhejiang province, while Yunnan Morus plant were collected from Jianshui, Yunnan province, China.

### Plant cold and chilling tolerance test

Stem cuttings of each varieties with three or four axillary buds were collected in winter and stored in 0 °C and −15 °C for 30 days, separately. The treated stems were then transferred to growth chamber (12/12 h day/light regime, 23 °C temperature, 200 µmol m^−2^ s^−1^ irradiances and 80% relative humidity) to calculated plant survival rates were for each variety. Regrown plants were scored as survivors.

The mulberry saplings of each varieties were produced from stem cuttings with three or four axillary buds and were grown for about 20 days at 27 °C with 12 h photoperiod of 200 µ mol m^−2^ s^−1^ in the growth chamber before the chilling test. Plants were then subjected to chilling stress at 2 °C for 48 h with a 12 h photoperiod. Each assay was conducted on at least three replications at a time with 10 individual plants per replicate.

### Electrolyte leakage assay

The electrolyte leakage assays were performed as described previously^35,36^. Briefly, the top 3^rd^ leaves of the plants subjected to the chilling tolerance test were collected, washed with deionized water, and placed into glass test tubes containing 10 ml of deionized water. One leave per accession was placed into individual test tubes, and three replicates per accession were tested. The tubes were shaken at 100 rpm for 2 h. The initial conductivity (Cini) was measured using an YSI3100 conductivity meter with an YSI 3253 Glass Dip Cell. The total conductivity (Ctot) was measured after boiling the samples for 10 min. Electrolyte leakage (EL) was calculated as

$${\rm{EL}}=({\rm{Cini}})/({\rm{Ctot}})\times 100.$$

### RNA extraction and gene expression assays

Leaf samples for gene expression analysis were collected after the seedling were treated under chilling conditions; the plants growing under normal conditions (25 °C with 12 h photoperiod of 200 µ mol m^2^ s^−1^) were collected and used as control. Total RNA was isolated from the leaves using TRIzol reagent (Invitrogen, Carlsbad, CA, USA).

For real-time quantitative PCR, first-strand cDNA synthesis was performed using the Reverse Transcription System (Tiangen, Beijing, China) with random primers according to the manufacturer’s instructions in a 20-μL reaction volume and an incubation time of 20 min at 42 °C from 0.1 μg total RNA. The cDNA reaction mixture was diluted two-fold with water, and 2 μL was used as a template in a 20-μL PCR on the Applied Biosystems StepOne Plus real-time PCR system in the standard mode using the SYBR Green PCR Core Reagents Mix (Applied Biosystems, California, USA). PCR was conducted after a preincubation at 95 °C for 3 min, followed by 40 cycles of denaturation at 95 °C for 3 s and extension at 55 °C for 30 s. Two biological and three technical replicates were performed for each experiment. *Actin* was used as the internal control for real-time quantitative PCR analysis. The abundance of transcripts was analyzed using the relative standard curve method normalized to the reference transcript *Actin*. The oligonucleotide primers used for amplification are shown in Supplemental Table 1.

### Protein extraction and West blot analysis

Total leaf proteins were extracted from the mulberry leaves with a protein extraction buffer (50 mM Tris, 150 mM NaCl, pH 7.5, 0.1% [v/v] Triton X-100, 0.2% [v/v] NP-40, 10 mM PMSF, and 5 mM EDTA). The crude extracts were centrifuged at 13,000 g for 30 min at 4 °C, and the supernatants were collected and added to an equal volume of 2 × SDS loading buffer (125 mM Tris-Cl, 2% SDS, 20% glycerol, 200 mM DTT, and 0.01% bromophenol blue, pH 6.8). About 50 μg protein per sample was loaded into each lane and separated on 10% SDS-PAGE gel for electrophoresis. Proteins from the gel were then transferred to a polyvinylidene difluoride membrane in a transfer buffer containing 20% methanol. After the transfer, nonspecific sites on the membrane were blocked with 5% (w/v) nonfat dry milk solution in TBST (0.1% Tween-20, 20 mM Tris base, 137 mM NaCl, and 3.8 mM HCl, pH 7.6) for 1 h, followed by incubation with the primary antibody for 2 h at room temperature. Antibodies against AKR2A (Shen, 2010), SOD (Agrisera, Vännäs, SWEDEN), FAD2 (Rabbit, polyclonal antibody) and KCS (Rabbit, polyclonal antibody) and Actin (Agrisera, Vännäs, SWEDEN) were used at a dilution of 1:1000. The blots were then washed three times with TBST prior to incubation with alkaline phosphatase–conjugated goat anti-rabbit antibodies (Bio-Rad) at a dilution of 1:5000 ratio for 1 h. The blot was then washed three times in TBST prior for color development with BCIP and NBT solutions (Bio-Rad).

### Histochemical detection of ROS

The production of hydrogen peroxide (H_2_O_2_) in leaves was detected by DAB staining method^37^. The leaves were submerged in 1 mg/ml DAB-HCl (pH 3.8), subjected to vacuum for 10 min, and incubated at room temperature for 3 h in the dark. In the presence of endogenous peroxidase, DAB was polymerized to brown DAB-polymer at the sites of H_2_O_2_ accumulation. For *in situ* detection of superoxide (${{\rm{O}}}_{2}^{\bullet -}$) the treated leaves were immersed in 10 mM K/Na phosphate buffer (pH 7.8) containing 0.1 mM NBT. The solution was vacuum infiltrated for 10 min. The leaves were then incubated at room temperature until the blue precipitates became visible^38^. Before capturing photographs, the treated whole leaves were cleared by boiling in ethanol: lactophenol: glycerol (3:1:1, v/v) for 10 min. SOD activity was determined in accordance with the instructions of total superoxide dismutase (T-SOD) assay kit (Jiancheng, Nanjing, China).

### Yeast two-hybrid and bimolecular fluorescence complementation (BIFC) assays

Y187 yeast competent cells were prepared according to the protocol of the Yeastmaker Yeast Transformation System (Clontech). Mulberry Y2H libraries (mulberry leaf library) were constructed according to the Make Your Own “Mate & Plate™” Library System User Manual (Clontech). To screen the proteins interacted with mAKR2A from the mulberry Y2H libraries, the protein mAKR2A with complete coding sequences was cloned into pGBKT7 (Clontech) and tested against auto-activity and toxicity according to the protocol, and mAKR2A (1–230 aa) was chosen for library screening. A concentrated Y2HGold (pGBDT7-mAKR2A) culture with 1 ml of the Y187 (pGADT7-mulberry library) Y2H library was mixed for mating in accordance with the Matchmaker™ Gold Yeast Two-Hybrid protocol (Clontech). DDO/X/A (lacking tryptophan and leucine supplemented with X-α-Gal and Aureobasidin A, Clontech) plates were used to screen the clones after mating for 120 h. All the colonies were then patched out and allowed to grow on QDO/X/A (lacking adenine, histidine, tryptophan and leucine and supplemented with X-α-Gal and Aureobasidin A. The bait plasmid (pGBKT7-53 or pGBKT7-Lam) was co-transformed into Y2HGold with the prey plasmid (pGADT7-T) to serve as a positive or negative control, respectively. The mulberry library inserts were further sequenced and analyzed using the MorusDB BLASTP (http://morus.swu.edu.cn/morusdb/blast) program.

The full-length sequence of mAKR2A, mFADII, mSOD1 and mKCS1 were amplified from the Zhejiang Morus cDNA library by PCR with Hifi DNA polymerase (Tiangen, Beijing, China). In the Yeast Two-Hybrid Assays, mAKR2A was used as the bait and mFADII, mSOD1 and mKCS1 was used as preys. The Yeast Two-Hybrid assays were performed as described previously^39,40^.

To generate the construct for the BiFC assay, the full length of mAKR2A, mFADII, mSOD1 and mKCS1 were subcloned into the pQBV3 vector, then recombined into the pCambia-NYFP and pCambia-CYFP vectors using the gateway system. The resulting constructs pCambia-NYFP-mFADII, pCambia-NYFP-mSOD1, pCambia-NYFP-mKCS1 and pCambia-CYFP-mAKR2A were transformed into GV3101, and the recombinant cells were transfected 30-day old tobacco leaves^41^. The transfected leaves were imaged using a Leica SP5 confocal microscope at 60 h post transfection.

### Fatty acid analyses

Total fatty acids were extracted from 1 g fresh tissue of the third or fourth leaf of the three mulberry varieties. The samples were dried at 60 °C and ground to a fine powder. Fatty acids were then extracted and subjected to transesterification by 0.4 M NaOH Methanol: Aether: mineral ether (1:1:1, v/v) at 25 °C for 15 h. Methylated fatty acids were determined by gas chromatography using the model Agilent 19091S-433. A C17 fatty acid (heptadecanoic acid; Sigma-Aldrich) was added before extraction to monitor the sample loss ratio and for quantitative purposes. All experiments were repeated three times^42,43^.

## Electronic supplementary material

Supplementary data

## References

[1] Chinnusamy V, Zhu JK, Sunkar R (2010). Gene regulation during cold stress acclimation in plants. Methods in molecular biology.

[2] He N (2013). Draft genome sequence of the mulberry tree Morus notabilis. Nature communications.

[3] Clement WL, Weiblen GD (2009). Morphological Evolution in the Mulberry Family (Moraceae). Systematic Botany.

[4] Hu X (2011). New isoprenylated flavonoids and adipogenesis-promoting constituents from Morus notabilis. Bioorganic & medicinal chemistry letters.

[5] Vijayan K, Doss SG, Chakraborti SP, Ghosh PD (2009). Breeding for salinity resistance in mulberry (Morus spp.). Euphytica.

[6] Kim DH (2015). Cytosolic targeting factor AKR2A captures chloroplast outer membrane-localized client proteins at the ribosome during translation. Nature communications.

[7] Kim DH (2011). Small heat shock protein Hsp17.8 functions as an AKR2A cofactor in the targeting of chloroplast outer membrane proteins in Arabidopsis. Plant physiology.

[8] Shen G (2010). ANKYRIN REPEAT-CONTAINING PROTEIN 2A is an essential molecular chaperone for peroxisomal membrane-bound ASCORBATE PEROXIDASE3 in Arabidopsis. The Plant cell.

[9] Bae W (2008). AKR2A-mediated import of chloroplast outer membrane proteins is essential for chloroplast biogenesis. Nature cell biology.

[10] Graham D, Patterson BD (1982). Responses of plants to low, nonfreezing temperatures: proteins. metabolism, and acclimation. Annual Review of Plant Physiology.

[11] Williams JP, Khan MU, Wong D (1992). Low temperature-induced fatty acid desaturation in Brassica napus: thermal deactivation and reactivation of the process. Biochimica et biophysica acta.

[12] Sakamoto T, Murata N (2002). Regulation of the desaturation of fatty acids and its role in tolerance to cold and salt stress. Current opinion in microbiology.

[13] Upchurch RG (2008). Fatty acid unsaturation, mobilization, and regulation in the response of plants to stress. Biotechnology letters.

[14] Palta JP, Whitaker BD, Weiss LS (1993). Plasma Membrane Lipids Associated with Genetic Variability in Freezing Tolerance and Cold Acclimation of Solanum Species. Plant physiology.

[15] Xue Y (2014). Arabidopsis membrane-associated acyl-CoA-binding protein ACBP1 is involved in stem cuticle formation. Journal of experimental botany.

[16] Vergnolle C (2005). The cold-induced early activation of phospholipase C and D pathways determines the response of two distinct clusters of genes in Arabidopsis cell suspensions. Plant physiology.

[17] Yan SP, Zhang QY, Tang ZC, Su WA, Sun WN (2006). Comparative proteomic analysis provides new insights into chilling stress responses in rice. Molecular & cellular proteomics: MCP.

[18] Shinozaki K, Yamaguchi-Shinozaki K, Seki M (2003). Regulatory network of gene expression in the drought and cold stress responses. Current opinion in plant biology.

[19] Li-Beisson Y (2013). Acyl-lipid metabolism. The Arabidopsis book.

[20] Shindou H, Eto M, Morimoto R, Shimizu T (2009). Identification of membrane O-acyltransferase family motifs. Biochemical and biophysical research communications.

[21] Hills, M. J. & Roscoe, T. J. *In The Plant Endoplasmic Reticulum* 155–186 (Springer, 2006).

[22] Sanchez R, Flores A, Cejudo FJ (2006). Arabidopsis phosphoenolpyruvate carboxylase genes encode immunologically unrelated polypeptides and are differentially expressed in response to drought and salt stress. Planta.

[23] Sanchez R, Cejudo FJ (2003). Identification and expression analysis of a gene encoding a bacterial-type phosphoenolpyruvate carboxylase from Arabidopsis and rice. Plant physiology.

[24] Martin DB, Vagelos PR (1962). The mechanism of tricarboxylic acid cycle regulation of fatty acid synthesis. The Journal of biological chemistry.

[25] Millar AA, Kunst L (1997). Very-long-chain fatty acid biosynthesis is controlled through the expression and specificity of the condensing enzyme. The Plant journal: for cell and molecular biology.

[26] Joubes J (2008). The VLCFA elongase gene family in Arabidopsis thaliana: phylogenetic analysis, 3D modelling and expression profiling. Plant molecular biology.

[27] Paul S (2006). Members of the Arabidopsis FAE1-like 3-ketoacyl-CoA synthase gene family substitute for the Elop proteins of Saccharomyces cerevisiae. The Journal of biological chemistry.

[28] Routaboul JM, Fischer SF, Browse J (2000). Trienoic fatty acids are required to maintain chloroplast function at low temperatures. Plant physiology.

[29] Shindou H, Shimizu T (2009). New development of lysophospholipid acyltransferases. Tanpakushitsu kakusan koso. Protein, nucleic acid, enzyme.

[30] Brown JK, Hovmoller MS (2002). Aerial dispersal of pathogens on the global and continental scales and its impact on plant disease. Science.

[31] Haas SE, Hooten MB, Rizzo DM, Meentemeyer RK (2011). Forest species diversity reduces disease risk in a generalist plant pathogen invasion. Ecology letters.

[32] Hartl FU, Hayer-Hartl M (2002). Molecular chaperones in the cytosol: from nascent chain to folded protein. Science.

[33] Wang W, Vinocur B, Shoseyov O, Altman A (2004). Role of plant heat-shock proteins and molecular chaperones in the abiotic stress response. Trends in plant science.

[34] Zhang H, Li X, Zhang Y, Kuppu S, Shen G (2010). Is AKR2A an essential molecular chaperone for a class of membrane-bound proteins in plants?. Plant signaling & behavior.

[35] Vogel JT, Zarka DG, Van Buskirk HA, Fowler SG, Thomashow MF (2005). Roles of the CBF2 and ZAT12 transcription factors in configuring the low temperature transcriptome of Arabidopsis. The Plant journal: for cell and molecular biology.

[36] Gilmour SJ, Fowler SG, Thomashow MF (2004). Arabidopsis transcriptional activators CBF1, CBF2, and CBF3 have matching functional activities. Plant molecular biology.

[37] Thordal-Christensen H, Zhang Z, Wei Y, Collinge DB (1997). Subcellular localization of H2O2 in plants. H2O2 accumulation in papillae and hypersensitive response during the barley—powdery mildew interaction. The Plant Journal.

[38] Fukao T, Yeung E, Bailey-Serres J (2011). The submergence tolerance regulator SUB1A mediates crosstalk between submergence and drought tolerance in rice. The Plant cell.

[39] Quint M, Ito H, Zhang W, Gray WM (2005). Characterization of a novel temperature‐sensitive allele of the CUL1/AXR6 subunit of SCF ubiquitin‐ligases. The Plant Journal.

[40] Wang S, Zheng H, Esaki Y, Kelly F, Yan W (2006). Cullin3 is a KLHL10-interacting protein preferentially expressed during late spermiogenesis. Biology of Reproduction.

[41] Lu Q (2010). Arabidopsis homolog of the yeast TREX-2 mRNA export complex: components and anchoring nucleoporin. The Plant journal: for cell and molecular biology.

[42] Okuley J (1994). Arabidopsis FAD2 gene encodes the enzyme that is essential for polyunsaturated lipid synthesis. The Plant cell.

[43] Craig W (2008). Transplastomic tobacco plants expressing a fatty acid desaturase gene exhibit altered fatty acid profiles and improved cold tolerance. Transgenic research.

