# Supplementary material for: The molecular chaperon AKR2A increases the mulberry chilling-tolerant capacity by maintaining SOD activity and unsaturated fatty acids composition
Source: Sci Rep. 2018 Aug 14;8:12120. doi: 10.1038/s41598-018-30379-9 (PMC6092340; doi:10.1038/s41598-018-30379-9)
Supplement: Supplementary file 1 — Supplementary data [file 41598_2018_30379_MOESM1_ESM.pdf]

The molecular chaperon AKR2A increases the mulberry chilling-tolerant capacity by maintaining SOD activity and unsaturated fatty acids composition

Lin Chen[1], Yuqi Hou[1], Wenjun Hu[1], Xiaoyun Qiu[1], Hongling Lu[1], Jia Wei[1], Shaofang Yu[1], NingJia He[3], Hong Zhang[2], Guoxin Shen[1]*

1 Sericultural Research Institute, Zhejiang Academy of Agricultural Sciences, Hangzhou 310021, China

2 Department of Biological Sciences, Texas Tech University, Lubbock, Texas 79409, USA

3 State Key Laboratory of Silkworm Genome Biology, Southwest University, Chongqing, 400715, China

*Corresponding author:

Guoxin Shen, Ph.D., Professor

Tel: +86-571-86404298; Fax: +86-571-86404298

Email address: guoxin.shen@ttu.edu

Supplemented data

Supplemented Table 1. Primers sequence

	Gene	Primer name	Sequence (5'-3')
qPCR			
	SOD1 (Morus011225)	qSOD1-F	GGAAGAACCTTGCTCCTATC
		qSOD1-R	ATGCTCCCAAACATCAATAC
	SOD2 (Morus010230)	qSOD2-F	CTGGTGACCTCGGAAACATC
		qSOD2-R	ATATCCAAATCCACGGTGCT
	SOD3 (Morus011779)	qSOD3-F	AAAGCCCTTCCAACTACAGA
		qSOD3-R	CAACAAGACGACGGTCCTAC
	LPAAT (Morus025929)	qLPAAT-F	CTCGACTCAAATCTGCTACAC
		qLPAAT-R	CACTCTTGCAGGTGTAAGAC
	PPC1 (Morus018660)	qPPC1-F	CCGACTCAAATCTGCTACAC
		qPPC1-R	CACTCTTGCAGGTGTAAGAC
	PPC4 (Morus025828)	qPPC4-F	CTCGACTCAAATCTGCTACAC
		qPPC4-R	CACTCTTGCAGGTGTAAGAC
	KCS1 (Morus014661)	qKCS1-F	CTCGACTCAAATCTGCTACAC
		qKCS1-R	CACTCTTGCAGGTGTAAGAC
	KAT1 (Morus017921)	qKAT1-F	CTCGACTCAAATCTGCTACAC
		qKAT1-R	CACTCTTGCAGGTGTAAGAC
	KAS1 (Morus021702)	qKAS1-F	CTCGACTCAAATCTGCTACAC
		qKAS1-R	CACTCTTGCAGGTGTAAGAC
	FADI (Morus018419)	qFADI-F	CTCGACTCAAATCTGCTACAC
		qFADI-R	CACTCTTGCAGGTGTAAGAC
	FADII (Morus025773)	qFADII-F	CTCGACTCAAATCTGCTACAC
		qFADI-R	CACTCTTGCAGGTGTAAGAC
	FADIII (Morus009908)	qFADIII-F	CTCGACTCAAATCTGCTACAC
		qFADI-R	CACTCTTGCAGGTGTAAGAC
	mAKR2A	qMAKR2A-F	CACTGCTCTACATTACGCAG

	(Morus013768)	qMAKR2A-R	ACAGGAAGGCATCCTTCTCG
	ACTIN (Morus007901)	qACTIN-F	GGGATGGGTCAGAAGGATGC
		qACTIN-R	CACTGGCGTAAAGGGAGAGAAC
**Y2H**			
	SOD1	SOD1ER1-F	GGAATTCATGGCTCTCCGAGCTCTCTTCC
		SOD1BH-R	CGGGATCCCTCGAGGGGGGTGTTCTTTTCG
	FADII	FADIIBH-F	CGGGATCCCGATGGCTCTGGTCACCTCACGACCATC
		FADIIXH-R	CCGCTCGAGGTTCAGAGCCATTCTTTGCTTCTGAGT C
	KCS1	KCS1ER1-F	GGAATTCATGCCTCCAATCTTGCCAGACTTCTC
		KCS1BH-R	CGGGATCCGAGCTTGACGATCTCCGGGATATGG
	mAKR2A$_{1-230}$	MAKR2ABH-F	CGGGATCCATGAGGATTAAGATGGCCCACAC
		MAKR2APS-R	AAAACTGCAGCCATGGCTGAGCAGCTTGGTAAC
**BIFC**			
	SOD1	QBSOD1-F	ATGGCTCTCCGAGCTCTCTTCC
		QBSOD1-R	CTCGAGGGGGGTGTTCTTTTCG
	FADII	QBFADII-F	ATGGCTCTGGTCACCTCACGACCATC
		QBFADII-R	GTTCAGAGCCATTCTTTGCTTCTGAGTC
	KCS1	QBKCS1-F	ATGCCTCCAATCTTGCCAGACTTCTC
		QBKCS1-F	GAGCTTGACGATCTCCGGGATATGG
	mAKR2A	QBmAKR2A-F	ATGAGGATTAAGATGGCCCACAC
		QBmAKR2A-R	CAGGAAGGCATCCTTCTCGAGCAAC

Supplemented data Figure. 1

A

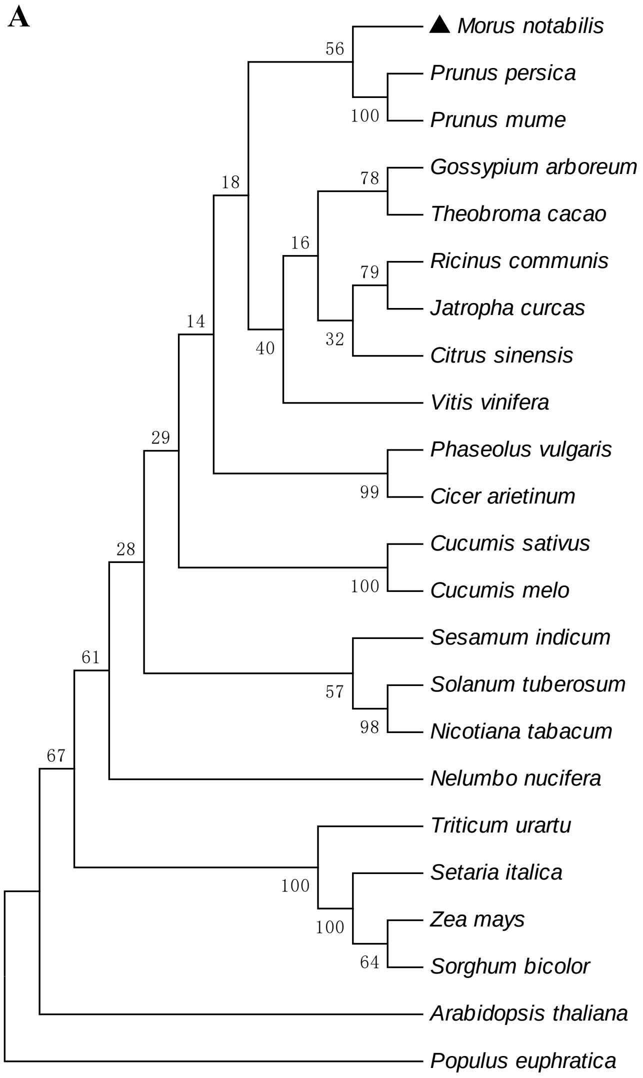

B

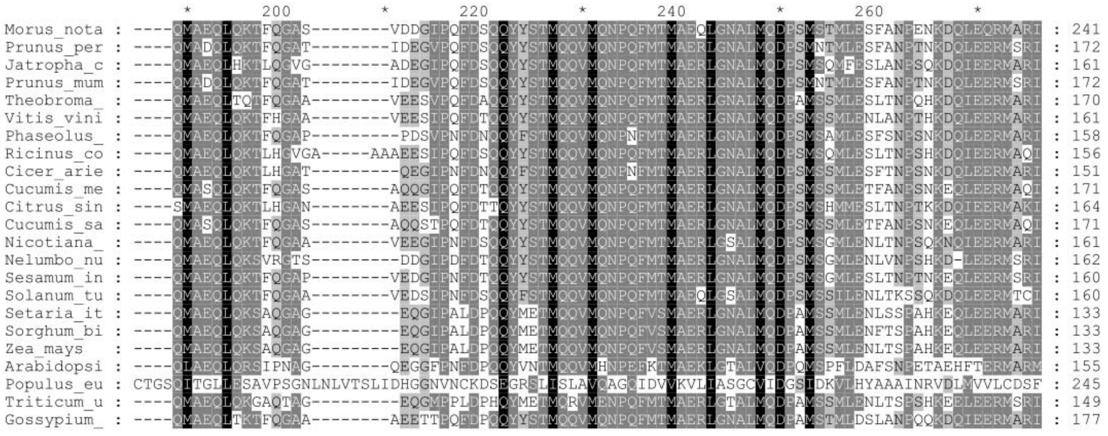

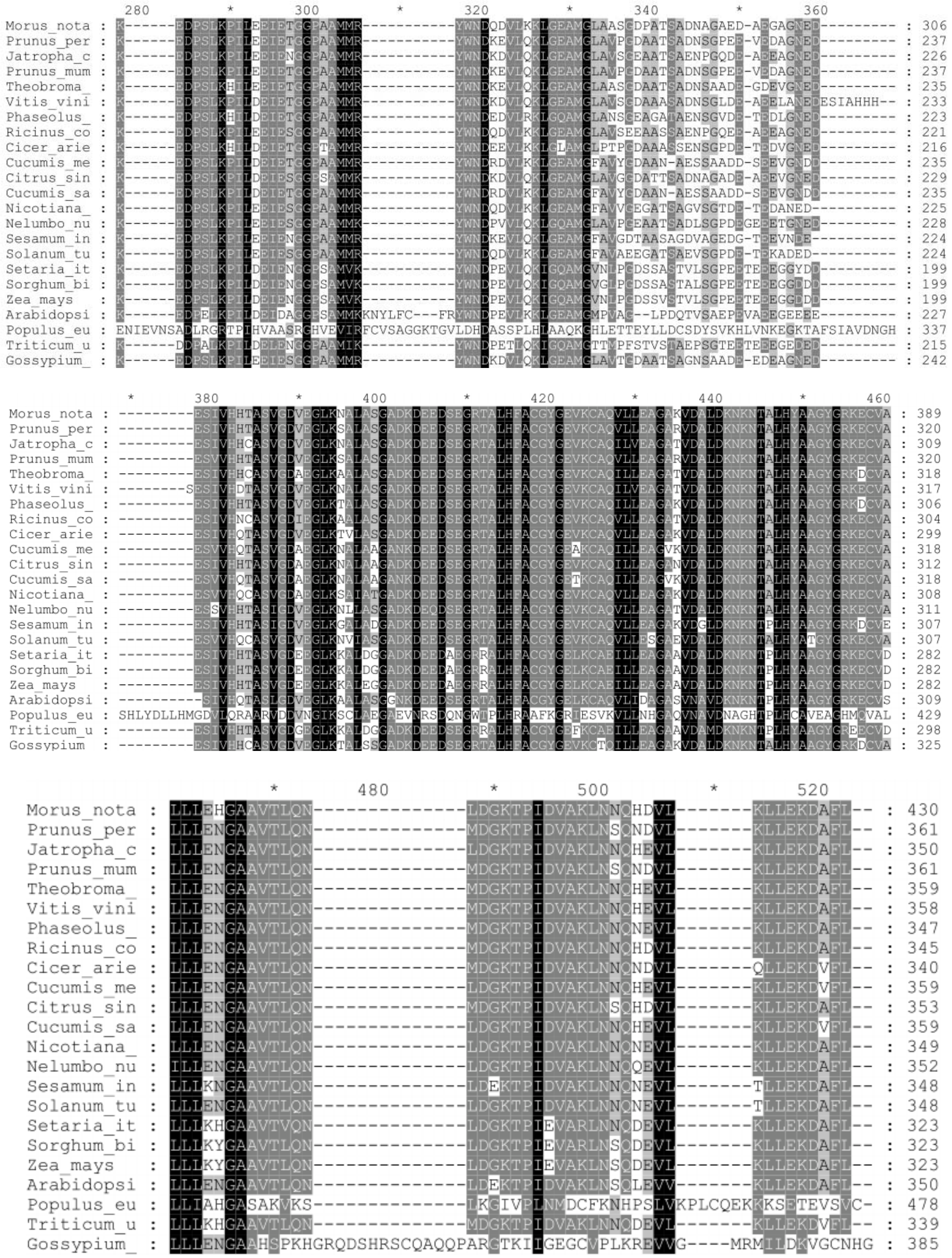

Supplemented data Figure. 1. (A) Multiple alignment of AKR2A homologues. (B) Conservation analysis of the AKR2A Ankyrin domain.

Supplemented data Figure. 2

A

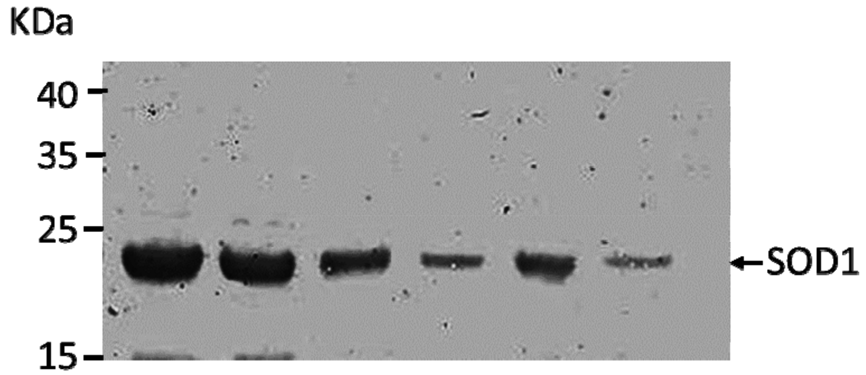

B

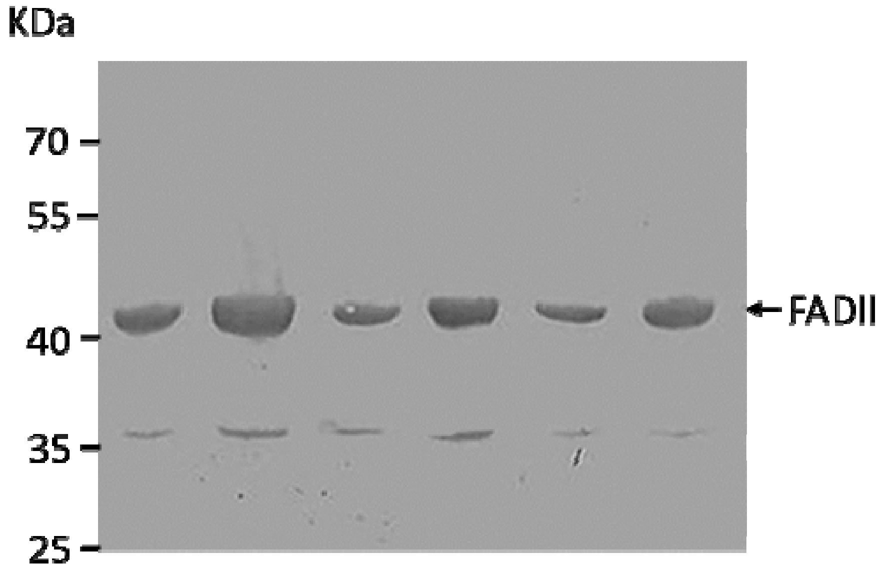

C

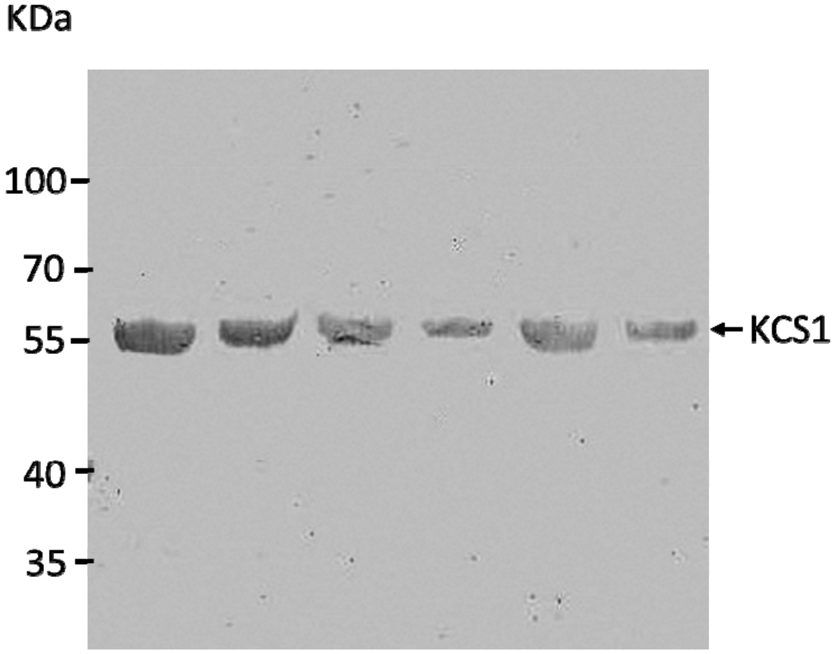

D

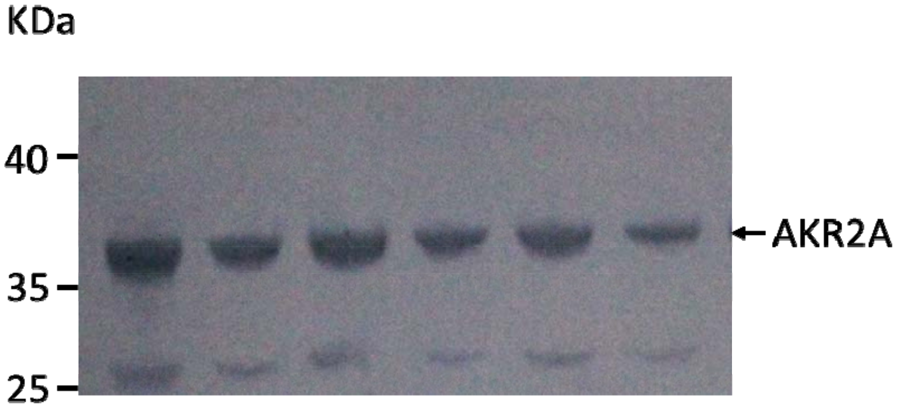

E

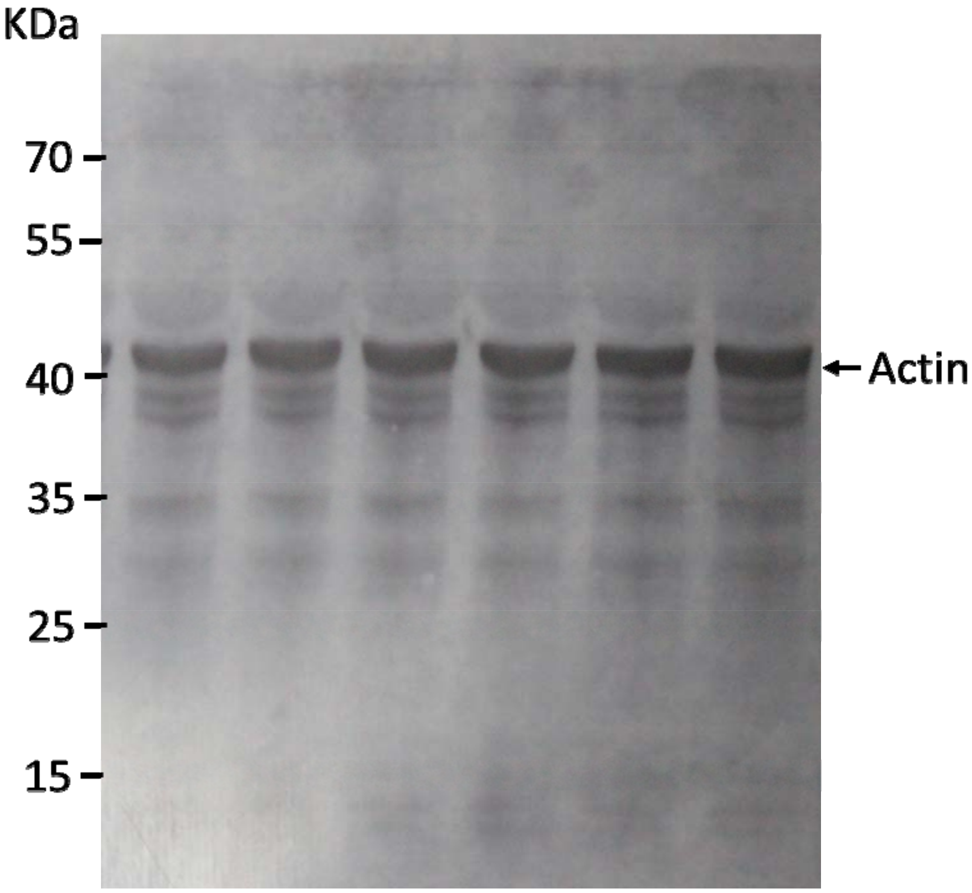

Supplemented data Figure. 2. Full-length blots of (A) SOD1, (B) FADII, (C) KCS1, (D) AKR2A, (E) Actin for Figure 5A.
